# Curcumin as an Epigenetic Modulator: Suppression of Breast Cancer via the Hsa_circ_0001946/MiR-7-5p/Target Gene Axis

**DOI:** 10.3390/medicina61091600

**Published:** 2025-09-04

**Authors:** Asmaa Abuaisha, Murat Kaya, Ilknur Suer, Selman Emiroglu, Aysel Bayram, Mustafa Tukenmez, Neslihan Cabioglu, Mahmut Muslumanoglu, Esra Nazligul, Berrin Papila, Abdulmelik Aytatlı, Omer Faruk Karatas, Kivanc Cefle, Sukru Palanduz, Sukru Ozturk

**Affiliations:** 1 Genetics Department, Institute of Graduate Studies in Health Sciences, Istanbul University, 34126 Istanbul, Turkey; 2 Biruni Research Center BAMER, Biruni University, 34015 Istanbul, Turkey; 3 Department of Internal Medicine, Division of Medical Genetics, Istanbul Faculty of Medicine, Istanbul University, 34093 Istanbul, Turkey; 4 Medical Genetics Department, Istanbul Faculty of Medicine, Istanbul University, 34093 Istanbul, Turkey; 5 Department of General Surgery, Division of Breast Surgery, Istanbul Faculty of Medicine, Istanbul University, 34093 Istanbul, Turkey; 6 Department of Molecular and Medical Genetics, Biruni University Graduate School of Education, 34015 Istanbul, Turkey; 7 Department of Medical Pathology, Surgical Medical Sciences, Istanbul Faculty of Medicine, Istanbul University, 34093 Istanbul, Turkey; 8 Department of Internal Medicine, Division of Hematology, Istanbul Faculty of Medicine, Istanbul University, 34093 Istanbul, Turkey; 9 Department of General Surgery, Cerrahpaşa Faculty of Medicine, Istanbul University-Cerrahpaşa, 34098 Istanbul, Turkey; 10 Department of Molecular Biology and Sciences, Faculty of Science, Erzurum Technical University, 25050 Istanbul, Turkey

**Keywords:** breast cancer, curcumin, Hsa_circ_0001946, CDR1as, MiR-7-5p, target genes, CKS2

## Abstract

*Background and Objectives*: Curcumin is a turmeric-derived polyphenol, and it has shown anticancer potential in various cancers, including breast cancer (BC). Nevertheless, the molecular mechanisms underlying its effects remain incompletely defined. Hsa_circ_0001946 (CDR1as) is a circular RNA (circRNA) that promotes tumor progression by competitively inhibiting microRNA-7-5p (miR-7-5p) in BC. This study investigated whether curcumin regulates the hsa_circ_0001946/miR-7-5p/target gene axis in BC progression. *Materials and Methods*: BC cell lines (MCF-7 and T47D) and a non-cancerous human mammary epithelial cell line (MCF-10A) were treated with curcumin or transfected with circ_0001946 siRNA or miR-7-5p mimic. Cell proliferation, migration, apoptosis, and protein expression were analyzed by CVDK-8 analysis, a wound healing assay, and flow cytometry, respectively. Also, protein expression levels were quantified via Western blotting. In vitro and in silico findings were further validated by analyzing tumor and adjacent normal tissues from 65 luminal BC patients. *Results*: Curcumin inhibited the proliferation and migration of MCF-7 and T47D cells in a dose-dependent manner. Knockdown of hsa_circ_0001946 or overexpression of miR-7-5p significantly suppressed proliferation and migration and enhanced apoptosis in BC cells compared to the negative controls. Curcumin treatment led to the knockdown of hsa_circ_0001946, the overexpression of miR-7-5p, and the downregulation of hsa_circ_0001946, *CKS2*, *TOP2A*, and *PARP1*, while it upregulating miR-7-5p. The Western blot confirmed reduced CKS2 protein levels after curcumin treatment. The expression of both hsa_circ_0001946 and *CKS2* was significantly upregulated in tumor tissues compared to that of matched adjacent normal tissues, whereas that of miR-7-5p was markedly downregulated. *Conclusions*: This preliminary study shows that curcumin suppresses BC tumorigenesis by modulating the hsa_circ_0001946/miR-7-5p/target gene axis. While these findings suggest a novel regulatory pathway and potential therapeutic targets, further in vivo validation and clinical trials are required to determine the translational relevance of curcumin in BC therapy.

## 1. Introduction

Breast cancer (BC) is the second most diagnosed cancer worldwide, and the most diagnosed cancer in women. According to the GLOBOCAN database in 2022, BC accounted 11.6% of all cancer incidences, and 6.9% of all cancer deaths [1]. BC is commonly grouped into four intrinsic molecular subtypes: luminal A, luminal B, HER2-positive and triple-negative breast cancer. Accurate classification is crucial for treatment and follow-up [2]. However, routine cancer treatments can cause drug-related side effects, toxicity and drug resistance with prolonged application. This highlights the necessity for more effective therapies or combination therapy approaches [3,4]. Due to resistance or severe side effects from standard BC treatments including chemotherapy and radiotherapy, many patients turn to herbal medicine due to their low toxicity and minimal side effects [5].

Curcumin is a natural polyphenol that is derived from the turmeric plant (*Curcuma longa* L.) and used as a spice and food additive [6]. Curcumin has been widely researched due to its anticancer, antioxidant, anti-inflammatory, antibacterial, and other pharmacological activities. Many studies have shown that curcumin exerts anticancer effects in various ways, inducing cell cycle arrest and apoptosis, modulating related signal pathways and gene expression, inhibiting tumor cell proliferation and metastasis, and suppressing angiogenesis [7,8,9]. Recent studies have suggested that curcumin can act as an epigenetic regulator in several cancers by regulating different non-coding RNAs (ncRNAs). Curcumin’s potential to alter ncRNA expression and target genes gives it the potential to be a new candidate for cancer treatment [10,11,12].

Circular RNAs (circRNAs) and microRNAs (miRNAs) are examples of the epigenetic modifications that influence the pathogenesis and progression of cancer [13]. CircRNAs are a large class of endogenous ncRNAs produced by the back-splicing of precursor RNAs to form a covalently closed-loop structure that lacks the 5′-cap and a 3′-poly(A) tail. CircRNAs play a crucial role as key regulators of gene expression by functioning as miRNA sponges, transcription modulators, or protein translation templates. Therefore, circRNAs can be oncogenic or tumor-suppressive, affecting multiple cellular processes such as cell proliferation, migration, invasion and apoptosis [14]. Hsa_circ_0001946 (also known as CDR1as and ciRS-7) is one of the most well-identified circRNAs that is involved in human tumorigenesis and that is dysregulated in different cancers, including BC [15]. Hsa_circ_0001946 promotes BC tumorigenesis and progression by competitively inhibiting miR-7-5p, which functions as a tumor suppressor miRNA, targeting several oncogenes [16,17,18,19]. Recent findings have highlighted the CDR1as/miR-7-5p/*IGF1R* axis as a promising molecular pathway with potential diagnostic and therapeutic implications in prostate cancer [20].

Curcumin’s ability to modulate miRNA expression has opened up new approaches in the field of cancer treatment [21,22]. In a recent study, curcumin was shown to inhibit the proliferation and migration of MCF-7 cells by regulating miR-145-5p and its possible target genes *MCM2*, *MMP1* and *MMP9* [23]. Another study demonstrated that curcumin exerts anticancer effects on MDA-MB-231 cells by modulating the miR-638-5p/*CFL1*, *SIX4* and *MAZ* genes axis [11]. In addition, it has been shown that curcumin treatment leads to a significant reduction in proteasome 20S subunit beta 5 (PSMB5) protein levels in TNBC cells. The same study reported that curcumin markedly increased the expression of miR-142-3p. Furthermore, curcumin was demonstrated to negatively regulate the levels of PSMB5 protein, one of the target genes of miR-142-3p [24]. Recently, several studies have explored the effects of curcumin through the regulation of circRNAs and miRNAs. Notably, however, no study to date has been identified that specifically investigates the relationship between curcumin and circRNAs in BC.

This study aimed to elucidate the functional role and biological effects of curcumin on the hsa_circ_0001946/miR-7-5p/target gene regulatory axis in MCF-7 and T47D breast cancer cell lines, using MCF-10A cells as a non-cancerous control. Additionally, we aimed to determine the relative expression levels of hsa_circ_0001946, miR-7-5p and the *CKS2* gene in tissue samples of 65 luminal A and luminal B BC patients. To the best of our knowledge, this is the first study to investigate the regulation of circRNAs by curcumin in BC, offering novel mechanistic insights and supporting its potential as a targeted therapeutic approach.

## 2. Materials and Methods

### 2.1. Tissue Specimens

This study was approved by the Ethical Committee of the Istanbul University, Istanbul Faculty of Medicine (File No. 2023/585; Date: 07.04.2023). Informed consent was obtained from all the patients before the procedure. Power analysis was performed to determine the sample size required for this study. The calculation method (d-value) developed by Cohen was used to calculate the size of the impact to be used in determining the sample size in the study [25]. The findings of Yang, W. et al. (2019) were used to determine the d value, which is the effect size index [18]. Based on the findings in this study, the effect size to be used in this research was calculated as d = 0.508. In this context, for the research hypotheses, it was envisaged that a total of 60 participants would be taken as a sample group using the G-power (version 3.1) package program with a d = 0.508, 99% confidence level (1-α) and 95% test power (1-β). Considering possible losses throughout the research, we decided to conduct the research with a total sample group of 65 patients.

BC tumor tissue and normal tissue adjacent to the tumor samples were collected from 30 luminal A- and 35 luminal B-subtype BC patients. All patients were women who had been diagnosed with invasive BC, with a tumor size of greater than 15 mm, who had not received chemotherapy or hormone therapy before being operated on in the Breast Surgery Division, General Surgery Department, Istanbul Faculty of Medicine of Istanbul University. All tissues to be used in this study were evaluated and obtained from the Pathology Department of Istanbul University. Tissue samples were washed with PBS and stored at −80 °C for RNA isolation. The samples were physically broken down by using a mortar and pestle and crushed in liquid nitrogen. Subsequently, the samples were resuspended in TRIzol Reagent (Invitrogen, USA) for total RNA isolation.

### 2.2. Cell Culture

The human luminal BC cell lines MCF-7 and T47D (ER+/HER2−) and the non-tumorigenic human mammary epithelial cell line MCF-10A (ER−) were utilized in this study [26,27]. MCF-7 cells were maintained in high-glucose Dulbecco’s Modified Eagle Medium (HG-DMEM) (EcoTech Biotechnology, Turkey) supplemented with 10% fetal bovine serum (FBS) (Gibco, UK) and 1% antibiotic (penicillin and streptomycin) (Gibco, USA). T47D cells were maintained in RPMI 1640 medium (EcoTech Biotechnology, Turkey) supplemented with 10% FBS and 1% antibiotic. MCF-10A cells were maintained in HG-DMEM medium supplemented with 10% FBS 1% antibiotic, 100 μg/mL epidermal growth factor (EGF) (Gibco, USA), 1 mg/mL hydrocortisone (Sigma-Aldrich, USA), 10 mg/mL insulin solution human (Sigma-Aldrich, USA) and MEM non-essential amino acid solution (100X) (Gibco, USA). Cultured cells were maintained at a humidity > 90% and 5% CO_2_ in a 37 °C incubator (SANYO, Japan).

### 2.3. Curcumin Treatment and Cell Transfection with circ_0001946 siRNA and miR-7-5p Mimic

Curcumin (Bio Basic Inc., Canada) was dissolved in 100% dimethyl sulfoxide (DMSO) (EcoTech Biotechnology, Turkey) at a concentration of 1 mg/mL. According to the recommendations in the previous literature, MCF-7 and T47D cells were treated with curcumin at different doses (5, 10, 20 μM) for 24 h [28,29]. DMSO was used as a solvent control. Curcumin was applied at a concentration of 10 μM for 24 h in the functional analysis, based on its IC_50_ value.

Small interfering RNA was used to knock down circ_0001946 (CDR1as siRNA). Silencer select negative control siRNA was used as a non-target siRNA negative control (NT siRNA) (Thermo Fisher Scientific, USA). The MCF-7 and T47D cell lines were transfected with 100 pmol of CDR1as siRNA and NT siRNA by using Lipofectamine 2000 transfection reagent (Invitrogen, USA) [30]. To investigate the functional role of miR-7-5p in BC cells, transfection experiments were performed using 100 pmol miR-7-5p mimic (Thermo Fisher Scientific, USA) and non-target miRNA mimic negative control (NT-miRNA) (MirVana-Ambion, USA) [31].

### 2.4. Cell Viability Detection

Cell viability detection kit 8 (CVDK-8) (EcoTech Biotechnology, Turkey) was employed to assess cells viability. Cells were cultured (3 × 10^3^ cells/well) into 96-well plates (Nest Biotechnology Co., China) and incubated under the suitable conditions. After 24 h, cells were treated or transfected and re-incubated. After 24 h, 10 μL of CVDK8 solution was added and the plates were incubated again. After 3 h, the absorbance was measured via a microplate reader (Thermo Fisher Scientific, USA) at 450 nm. The cell morphology and cell proliferation state were evaluated by the viability imaging method as described by Kaya et al. [11]. The plates were washed with phosphate-buffered saline (PBS), and a phase-contrast microscope (Nikon, Japan) was used to capture morphology images.

### 2.5. Wound Healing Assay

The scratch assay was used for cell migration detection. Cells were cultured (4 × 10^5^ cells/well) into six-well plates (Nest Biotechnology Co., China) and incubated under the suitable conditions. Once the cells reached near confluency, the monolayer was scratched using a sterile pipette tip. The culture medium was then removed, and the cells were gently washed twice with PBS to eliminate detached cells and debris. After washing, 2 mL of fresh medium containing the indicated treatment or transfection reagents was added to each well. By using a phase-contrast microscope, the wound area was recorded at 0 and 24 h and the relative migration rate was calculated in accordance with the protocol followed by Abuaisha et al. [23].

### 2.6. Cell Apoptosis Analysis

Cell apoptosis was evaluated by using the Annexin V-FITC apoptosis detection kit (eBioscience-Invitrogen, Austria). MCF-7 cells were seeded (4 × 10^5^ cells/well) into six-well plates and incubated. After 24 h, the cells reached approximately 60% confluency and were transfected with circ_0001946 siRNA, miR-7-5p mimic, or their respective negative controls. After 48 h, the cells were harvested and washed three times with cold PBS. After that, a 195 μL cell suspension was incubated with the 5 μL Annexin V-FITC for 15 min at room temperature in the dark. Afterwards, the cells were washed with 200 μL of binding buffer (1x) and resuspended in 190 μL of binding buffer (1x). Next, 2.5 μL of propidium iodide (20 μg/mL) was added and incubated in the dark for 15 min [32]. The apoptosis rate of the cells was then measured using a Beckman Coulter Navios flow cytometer (Navios 3L10, Beckman Coulter, USA). The results were analyzed in the Kaluza analysis program.

### 2.7. Identification of the Potential Target Genes of miR-7-5p

Potential target genes of miR-7-5p were first searched for in the miRNet database by selecting the options “human”, “miRBase ID” and “miRTarBase V9” and protein–protein interaction (PPI) was marked. Afterwards, a search was made by typing “hsa-miR-7-5p”. In parallel, the TCGA-Breast Cancer dataset in the https://www.cancer.gov/ccg/research/genome-sequencing/tcga (accessed on 15 July 1015) database was downloaded and reanalyzed using GEO2R. Genes that were significantly upregulated in BC tissue samples were determined based on the criteria of LogFC > 1.5 and *p* < 0.01. Subsequently, ten overlapping genes between the TCGA breast cancer dataset and the miRNet dataset were identified. To evaluate the interactions among the identified genes, the STRING database [33] was used. The official gene names were input into the search field, and interaction networks were generated accordingly.

### 2.8. Relative Expression Levels Evaluated by qPCR

The relative expression of hsa_circ_0001946 and the identified genes were evaluated using quantitative real-time polymerase chain reaction (qPCR). Total RNA isolation was performed using TRIzol Reagent (Invitrogen, USA) in accordance with the manufacturer’s protocol. The concentration and purity of the isolated RNA were assessed using a NanoDrop ND-2000c spectrophotometer (Thermo Fisher Scientific Inc., USA). OneScript^®^ Plus cDNA Synthesis Kit (Applied Biological Materials Inc. Abm, Canada) was used for cDNA synthesis by utilizing 1000 ng total RNA in the T100 thermal cycler PCR machine (Bio-Rad, Singapore). qPCR experiments were performed in duplicate by using the BlasTaq 2X qPCR Master Mix kit (Abm, Canada) and the LightCycler 480 instrument (Roche, Germany). *GAPDH* was used to normalize gene expression data. For miRNAs, cDNA synthesis was achieved by using TaqMan™ MicroRNA Reverse Transcription Kit (Applied Biosystems, Thermo Fisher, Lithuania), the hsa-miR-7-5p TaqMan™ MicroRNA Assay S primer (005723_mat, hsa-miR-7-5p) (Applied Biosystems, Lithuania) and 100 ng/μL total RNA. RNU43 was used as a normalization control to standardize the expression of miRNA, and the TaqMan™ MicroRNA Assay S RNU43 primer (001095) (Applied Biosystems, Lithuania) was used for cDNA synthesis. All experiments were performed in duplicate using the TaqMan miRNA probes and the TaqMan™ Universal Master Mix Kit from Applied Biosystems, Thermo Fisher Scientific Baltics UAB, Lithuania. The qPCR reaction was performed as previously outlined by Abuaisha et al. [23] utilizing the LightCycler 480 instrument.

### 2.9. Western Blot Analysis

MCF-7 and T47D cells were seeded in duplicates into 6-well plates. After 48 h of curcumin treatment, the cells were harvested and washed twice with ice-cold PBS. Cell lysis was performed using RIPA buffer (EcoTech Biotechnology, Turkey) supplemented with 10 mM phenylmethylsulfonyl fluoride (PMSF, Roche) and a protease inhibitor cocktail (Thermo Fisher Scientific, USA). The cell lysate was centrifuged at 14.000 g for 15 min at 4 °C to separate cell debris and collect the supernatant containing the cellular proteins. The total protein concentrations were quantified using ClearBand Bradford Reagent (EcoTech Biotechnology, Turkey). After cell lysates were mixed with 10X Laemmli Sample Buffer (EcoTech Biotechnology, Turkey), the samples were boiled at 100 °C for 5 min. Subsequently, equal amounts of protein (30 μg) were separated on 10% SDS-PAGE gel and transferred to a nitrocellulose membrane (EcoTech Biotechnology, Turkey) using the Semi-Dry Trans-Blot Turbo Transfer System (Bio-Rad, Hercules, CA, USA). Membranes were blocked with 5% skimmed milk for 1 h and washed three times with 1X PBST buffer (EcoTech Biotechnology, Turkey) for 20 min. Afterwards, the membranes were incubated overnight at 4 °C with PBST-diluted primary antibodies against CKS2 (Thermo Fisher, USA) (100 UL) and β-actin (Santa Cruz Biotechnology, USA). After washing with PBST buffer for 20 min at least twice, HRP-conjugated anti-rabbit and anti-mouse secondary antibodies (Santa Cruz Biotechnology, USA) were applied for 1 h at 25 °C. Protein signals were visualized using the ClearBand ECL Western Blotting Substrate (EcoTech Biotechnology, Turkey). Quantification of the specific protein bands was performed with ImageJ 1.53f software [34].

### 2.10. Statistical Analysis

The normal distribution of continuous variables was examined with the Kolmogorov–Smirnov test. Categorical variables were presented as the frequency (*n*%), and continuous variables as the mean and standard deviation. All statistical calculations were performed with SPSS software version 26 (IBM Corp., Armonk, NY, USA). Significance was assessed at a 95% confidence interval, with a threshold of *p* < 0.05. The 2^−ΔΔCt^ method was used in the relative quantitation analysis of qPCR results. Student’s *t*-test was applied to compare the study group and the control group. *p* values were provided as the mean ± standard deviation, and expressed as * *p* < 0.05, ** *p* < 0.01, *** *p* < 0.001, indicating statistical significance. GraphPad Prism 10 software was used for graphic drawings and for the editing of the figures.

## 3. Results

### 3.1. Curcumin Inhibits the Proliferation and Migration of BC Cells

**Figure 1:**
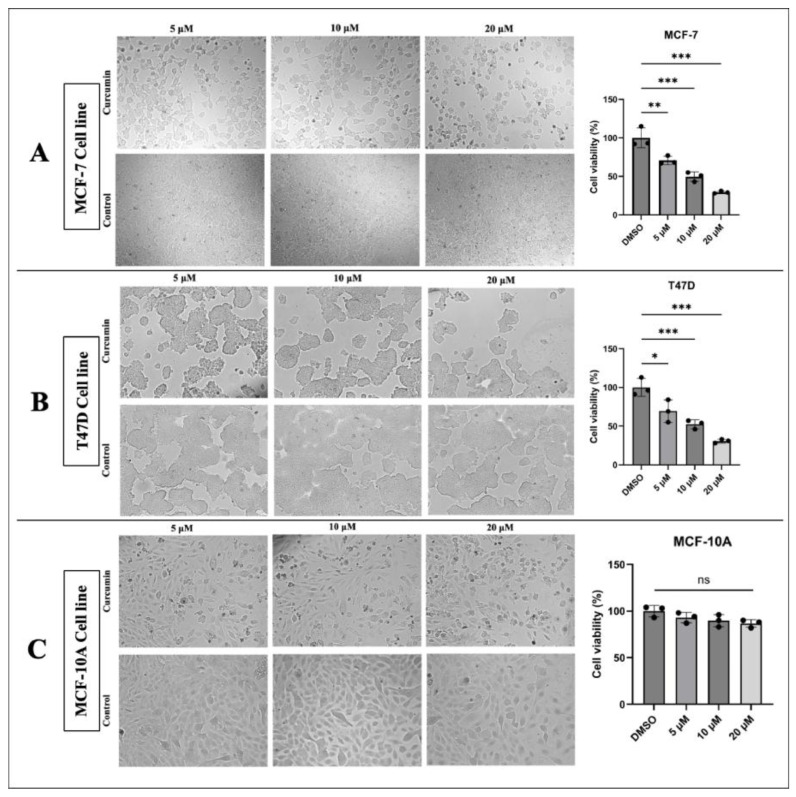
Cell viability and cell proliferation state. Curcumin treatment reduced cell viability and proliferation in a dose-dependent manner in (**A**) the MCF-7 cell line, and (**B**) the T47D cell line. (**C**) Curcumin caused a non-significant change in the viability and proliferation of MCF-10A cells (* *p* < 0.05, ** *p* < 0.01, *** *p* < 0.001). Error bars represent the standard deviations.

Significantly, curcumin treatment decreased the cell viability and proliferation state depending on increasing concentrations in MFC-7 and T47D cell lines. The non-cancerous MCF-10A cell line showed a non-significant change in viability and proliferation after curcumin treatment (Figure 1).

**Figure 2:**
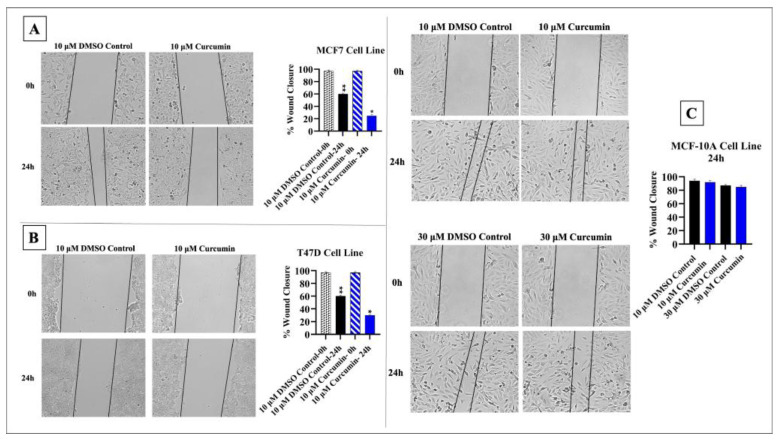
Effects of curcumin treatment on cell migration. (**A**) Curcumin inhibited the migration of MCF-7 cells. (**B**) Curcumin reduced the migration of T47D cells. (**C**) Curcumin had a minimal and non-significant effect on MCF-10A cells (* *p* < 0.05, ** *p* < 0.01). Error bars represent the standard deviations.

The half-maximal inhibitory concentration (IC_50_) value of curcumin was 10 μM, and therefore, this concentration was used in the following experiments. Cells’ treatment with 10 μM curcumin significantly inhibited the migration of MCF-7 and T47D cells compared to that of the controls (*p* < 0.05). In MCF-10A cells, 10 μM and 30 μM curcumin treatment had a quite limited effect on cell migration compared to the effect seen in cancer cells (Figure 2).

### 3.2. Curcumin Alters Expression of hsa_circ_0001946 and miR-7-5p in BC Cell Line

**Figure 3:**
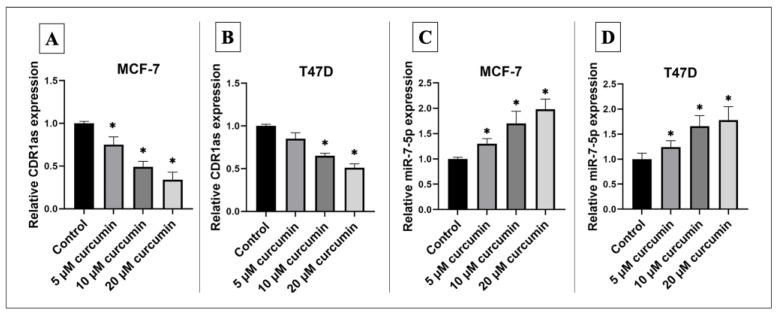
Relative expression level of hsa_circ_0001946 and miR-7-5p after curcumin treatment. (**A**) Curcumin treatment significantly downregulated hsa_circ_0001946 expression in MCF-7 cells. (**B**) A similar downregulation of hsa_circ_0001946/CDR1as was observed in T47D cells following curcumin treatment. (**C**) MiR-7-5p expression was significantly upregulated in MCF-7 cells after curcumin treatment. (**D**) Curcumin induced a significant upregulation of miR-7-5p in T47D cells (* *p* < 0.05). Error bars represent the standard deviations.

Treatment of MCF-7 and T47D cells with curcumin resulted in a significant downregulation of hsa_circ_0001946/CDR1as expression alongside a significant upregulation of miR-7-5p expression in a dose-dependent manner (Figure 3).

### 3.3. Knockdown of circ_0001946 Hindered the Viability and Migration of BC Cells by Upregulating miR-7-5p

**Figure 4:**
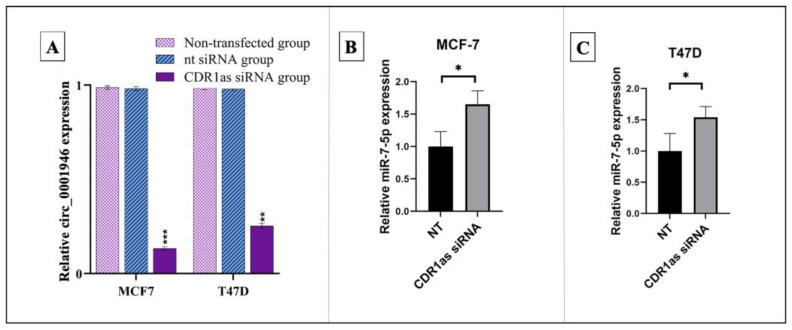
(**A**) Validation of CDR1as siRNA transfection. (**B**) Relative expression levels of miR-7-5p after CDR1as siRNA and NT siRNA control transfection in MCF-7 cell line. (**C**) MiR-7-5p expression after CDR1as siRNA and NT siRNA control transfection in T47D cell line (* *p* < 0.05, ** *p* < 0.01, *** *p* < 0.001) (NT: non-target siRNA negative control). Error bars represent the standard deviations.

Successful transfection of CDR1as siRNA into MCF-7 and T47D cell lines resulted in a significant reduction in circ_0001946 expression compared to the control group. Correspondingly, the relative expression level of miR-7-5p was significantly increased in both cell lines following CDR1as siRNA transfection (Figure 4).

**Figure 5:**
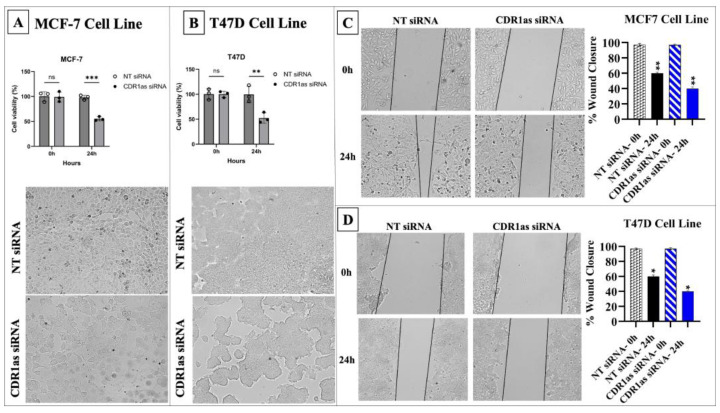
Circ_0001946 knockdown inhibited viability and migration in MCF-7 and T47D cells. (**A**) Microscopic images of MCF-7 cells after CDR1as siRNA transfection. (**B**) Microscopic images of T47D cells after CDR1as siRNA transfection. (**C**) Migration assay results for MCF-7 cells. (**D**) Migration assay results for T47D cells (* *p* < 0.05, ** *p* < 0.01, *** *p* < 0.001). Error bars represent the standard deviations.

Cell viability, proliferation, and migration were significantly reduced in MCF-7 and T47D cells following transfection with CDR1as siRNA compared to NT siRNA (*p* < 0.05) (Figure 5).

**Figure 6:**
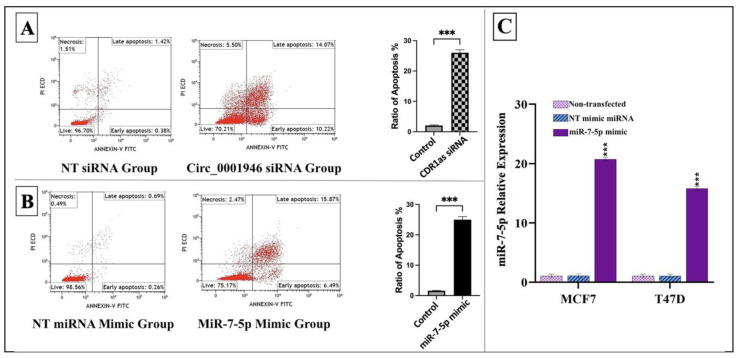
Effects of circ_0001946 knockdown and miR-7-5p overexpression on apoptosis and miR-7-5p expression. (**A**) Cell apoptosis rates following circ_0001946 knockdown in MCF-7 cells. (**B**) Cell apoptosis rates following miR-7-5p overexpression in MCF-7 cells. (**C**) Validation of miR-7-5p mimic transfection in MCF-7 and T47D cell lines (*** *p*  <  0.001). Error bars represent the standard deviations.

Moreover, successful transfection of the miR-7-5p mimic into MCF-7 and T47D cells resulted in a significant increase in miR-7-5p expression compared to that in the control group. Apoptotic cell rates were notably elevated in MCF-7 cells transfected with either CDR1as siRNA or the miR-7-5p mimic, as shown in Figure 6.

**Figure 7:**
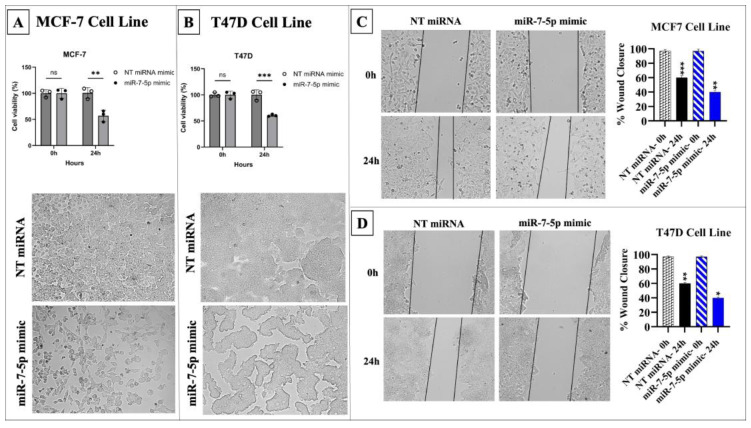
MiR-7-5p overexpression reduced the viability and migration of MCF-7 and T47D cells. (**A**) Microscopic images of MCF-7 cells after miR-7-5p mimic transfection. (**B**) Microscopic images of T47D cells after miR-7-5p mimic transfection. (**C**) Migration assay results for MCF-7 cells. (**D**) Migration assay results for T47D cells (* *p* < 0.05, ** *p* < 0.01, *** *p* < 0.001). Error bars represent the standard deviations.

Additionally, cell viability, proliferation, and migration significantly decreased in MCF-7 and T47D cells transfected with the miR-7-5p mimic compared to the control (*p* < 0.05) (Figure 7).

### 3.4. Bioinformatic Analysis of miR-7-5p Target Genes

**Figure 8:**
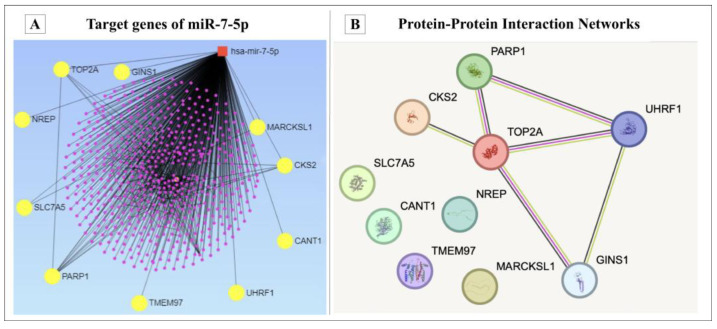
Bioinformatic analysis results of miR-7-5p target genes in BC. (**A**) The miR-7-5p in silico target genes. The red square is miR-7-5p. Yellow nodes involve 578 genes, including *CKS2, TOP2A, GINS1, NREP, SLC7A5, PARP1, TMEM97, UHRF1, CANT1* and *MARCKSL1*. (**B**) STRING analysis results: nodes: 10; edges: 6; average node degree: 1.2; avg. local clustering coefficient: 0.4; expected edges: 1; PPI enrichment *p*-value: 0.000567.

MiRNet analysis identified 578 potential target genes for miR-7-5p, with 1023 predicted interactions among them. According to the analysis of TCGA BC data, 545 genes were found to be upregulated in BC samples under the defined selection criteria. In total, 10 overlapped genes between TCGA data and miRNet data were identified for this study. The selected genes were *CKS2, TOP2A, GINS1, NREP, SLC7A5, PARP1, TMEM97, UHRF1, CANT1* and *MARCKSL1*, as presented in Figure 8. Using the STRING database, a stronger relationship was observed in the potential PPI analysis between the *CKS2*, *PARP1*, *TOP2A*, *UHRF1* and *GINS1* genes, and the PPI enrichment *p*-value was below 0.000567 (Figure 8).

### 3.5. Curcumin Suppresses BC Cells by Modulating miR-7-5p Target Genes

The expression levels of the selected genes (*CKS2, TOP2A, GINS1, NREP, SLC7A5, PARP1, TMEM97, UHRF1, CANT1* and *MARCKSL1*) were determined using the qPCR technique. The forward and reverse primer sequences employed in this study are listed in Table 1.

**Figure 9:**
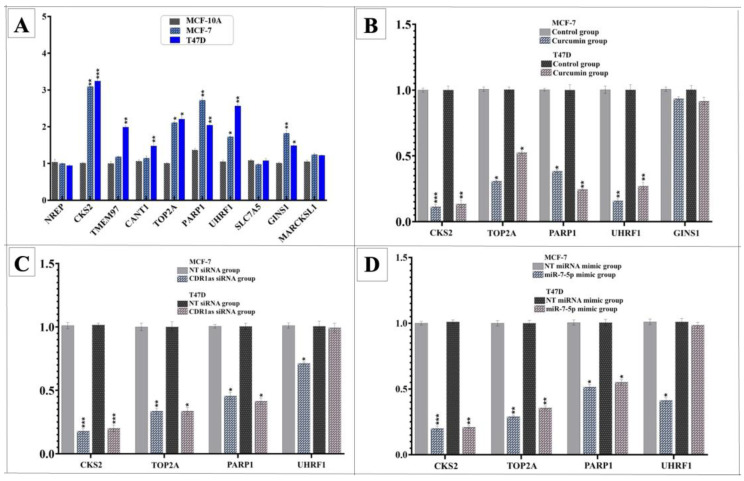
Relative expression levels according to qPCR and Western blot analysis results. (**A**) Relative expression levels of selected genes between BC cells and MCF-10A. (**B**) Gene expression changes in MCF-7 and T47D cells following curcumin treatment. (**C**) Expression levels of the selected genes after transfection with CDR1as siRNA. (**D**) Expression levels of the selected genes after transfection with the miR-7-5p mimic (* *p* < 0.05, ** *p* < 0.01, *** *p* < 0.001). Error bars represent the standard deviations.

To identify potential curcumin-regulated targets, gene expression profiles of BC cell lines were first compared with those of the MCF-10A cell line. Five genes (*CKS2, TOP2A, PARP1, UHRF1*, and *GINS1*) were significantly upregulated in both the MCF-7 and T47D cell lines. Among these, treatment with curcumin led to a significant downregulation of *CKS2, TOP2A, PARP1,* and *UHRF1* in both BC cell lines. Transfection experiments with CDR1as siRNA or the miR-7-5p mimic further confirmed that the expression of all four genes was significantly reduced in MCF-7 cells, and that the expressions of three of these genes (*CKS2, TOP2A,* and *PARP1*) were similarly downregulated in T47D cells (Figure 9).

**Figure 10:**
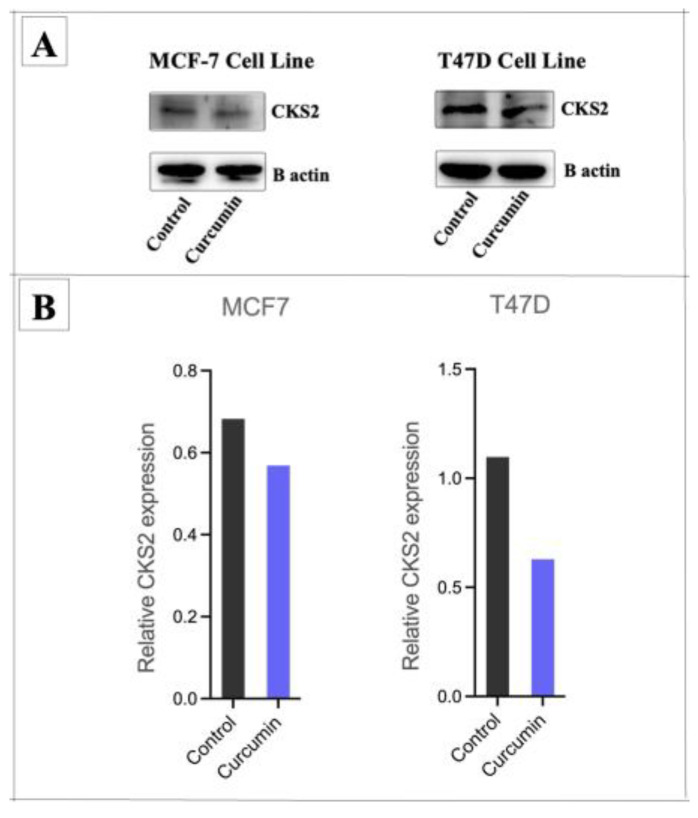
Western blot analysis showing reduced CKS2 protein expression in MCF-7 and T47D cells after curcumin treatment. (**A**) Representative images of Western blot analysis of CKS2, and with B actin as the loading control in MCF-7 and T47D cells. (**B**) Curcumin treatment significantly decreased CKS2 protein expression in both the MCF-7 and T47D cell lines.

Among the candidates, *CKS2* was selected for validation at the protein level. Western blot analysis demonstrated that curcumin treatment significantly decreased CKS2 protein expression in both MCF-7 and T47D cell lines (Figure 10).

### 3.6. Hsa_circ_0001946/miR-7-5p/CKS2 Axis as Potential Biomarker in Breast Cancer 

The demographic, and clinicopathological characteristics of the patients are presented in Table 2 and Table 3. All the patients were women. In total, 30 patients (46.2%) had the luminal A subtype of BC, and 35 patients (53.8%) had the luminal B subtype. The average age of the patients was 56. In total, 69.2% of the patients were in the postmenopausal period, 24.6% had a family history of BC, 20% were smokers, and 4.6% consumed alcohol. All patients were ER-positive, 90.8% were PR-positive, and 9.2% were HER2-positive. The histological type of 72.3% of the tumors was invasive ductal carcinoma. The pathological tumor stage was T2 in 70.8% of the patients. Lymph node involvement was present in 41.5% of the patients, and lympho-vascular invasion was present in 60% of the patients.

**Figure 11:**
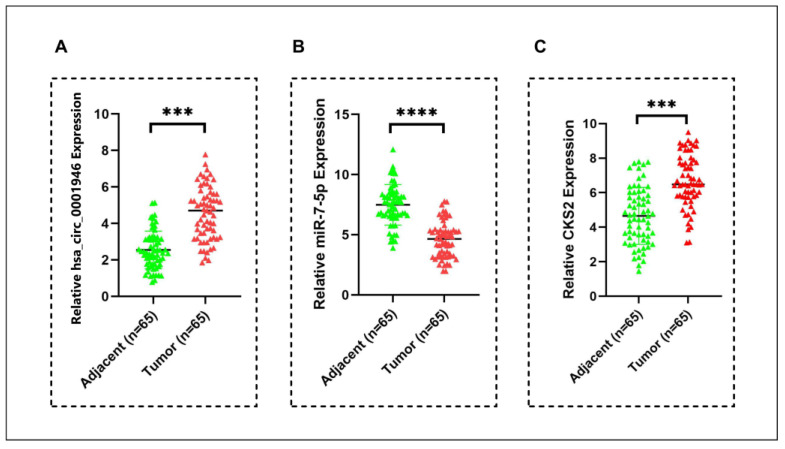
Relative expression levels of hsa_circ_0001946, miR-7-5p and CKS2 in BC patient tumor tissue samples compared to adjacent normal tissue samples. (**A**) The expression level of hsa_circ_0001946 was significantly upregulated in tumor tissues. (**B**) MiR-7-5p expression was significantly decreased in breast cancer samples. (**C**) *CKS2* expression was consistently upregulated in BC tissues (*** *p* < 0.001, **** *p* < 0.0001). Error bars represent the standard deviations.

The expression level of miR-7-5p was significantly reduced in cancer tissue samples. On the contrary, hsa_circ_0001946 and *CKS2* expression was significantly increased in BC tissue samples (Figure 11). These findings are consistent with the results of the in silico analysis and cell culture experiments.

## 4. Discussion

Breast cancer is the most common malignancy among women and is characterized by both genetic and epigenetic alterations that disrupt multiple signaling pathways. Due to its heterogeneous nature and the influence of tumor microenvironmental factors, BC cells often develop resistance to therapy. As a result, targeting a single signaling pathway is typically insufficient for effective treatment [46]. This highlights the importance of developing improved therapies and exploring combination approaches. In the United States, approximately 50–60% of cancer patients use natural therapies either as complementary or alternative approaches to conventional treatments, primarily due to their low toxicity and minimal side effects [5].

Curcumin, derived from *Curcuma longa*, has gained attention for its antioxidant, anti-inflammatory, and anticancer properties, showing potential in managing cancer, as well as metabolic and neurodegenerative diseases [47]. In recent years, numerous in vitro and in vivo studies have demonstrated that curcumin, a natural polyphenol extracted from the turmeric plant, exhibits therapeutic potential against various diseases, including BC [11,23,24]. It exerts multifaceted anticancer effects by modulating signaling pathways, suppressing cell proliferation, inducing apoptosis, and promoting reactive oxygen species production [48]. Over the past 20 years, the large number of in vitro and preclinical studies has led to a significant increase in clinical trials investigating the therapeutic potential of curcumin-containing products. In these clinical studies, various formulations and curcuminoid doses have been tested for cancer prevention and treatment, as well as for improving cancer therapy-related symptoms [49]. More than 50 clinical trials of curcumin-based therapeutics targeting cancer have been completed [50]. Examples include a Phase I clinical trial of curcumin monotherapy in BC patients (NCT03980509) and a Phase II clinical trial combining curcumin with paclitaxel (NCT03072992). In addition to BC patients, a Phase II clinical trial involving cervical and uterine cancer patients (NCT03192059) has also been completed, while a Phase III clinical trial involving prostate cancer patients (NCT03769766) is still ongoing [51,52]

In addition to the phytotherapy, emerging epigenetic therapeutics, including mRNA regulators (such as miRNA mimics and antagomiRs), DNA methyltransferase (DNMT) inhibitors, and histone-modifying enzymes, have shown promise in reversing these aberrant alterations, positioning them as potential candidates for cancer therapy [53]. DNMT inhibitors like 5-azacytidine and decitabine (5-aza-2′-deoxycytidine) have already been approved by the FDA for the treatment of acute myeloid leukemia and myelodysplastic syndrome when used in combination with conventional therapies [54]. Similarly, the future development of drugs targeting circRNA/miRNA-based epigenetic regulation may offer a novel and effective strategy for the treatment of BC [53,55].

The circRNA/miRNA/mRNA regulatory axis may play a critical role in cancer development, progression, and therapeutic response [56]. Given the existence of thousands of circRNAs, the ability of a single circRNA to act as a sponge for multiple miRNAs, and the capacity of a single miRNA to regulate hundreds of target genes, it is evident that the current literature provides only a limited understanding of the complex relationship between circRNAs and BC. Moreover, studies investigating the potential of circRNAs to improve the classification and treatment of BC remain scarce [57]. Recent studies have shown that the anticancer effects of curcumin may be attributed to the regulation of the circRNA/miRNA/mRNA pathway [58,59]. In this study, we investigated, for the first time, the potential antitumor effects of curcumin through its modulation of the hsa_circ_0001946/miR-7-5p/target gene axis, a regulatory pathway implicated in BC progression.

The results of this study demonstrated that the expression levels of hsa_circ_0001946 were upregulated in both BC cell lines and patient tissue samples, consistent with the results of previous studies [15,60]. Conversely, miR-7-5p was downregulated, and this is in agreement with findings in the literature [18,19]. Moreover, the oncogenic targets of the miR-7-5p, *CKS2*, *TOP2A*, *PARP1*, *GINS1* and *UHRF1* genes were upregulated, supporting the role of hsa_circ_0001946 as a miRNA sponge regulating downstream gene expression. To our knowledge, this is the first study to investigate the effects of curcumin mediated by a circRNA in BC. Here we showed the effects of curcumin on the hsa_circ_0001946/miR-7-5p/target genes axis in inhibiting BC progression. Curcumin dose-dependently restricted the proliferation and migration of BC cells, in addition to decreasing the expression of hsa_circ_0001946 and increasing that of miR-7-5p. Hsa_circ_0001946 knockdown and miR-7-5p upregulation induced apoptosis and reduced proliferation and migration in BC cells. In a study conducted on colorectal cancer cells, curcumin reduced the aggressiveness of cells by acting via the circHN1/miR-302a-3p/*PIK3R3* pathway [59]. Another study reported that curcumin inhibited cell proliferation and increased apoptosis in ovarian cancer by regulating the circ-PLEKHM3/miR-320a/*SMG1* axis [58]. Additionally, curcumin increases radiotherapy sensitivity in nasopharyngeal cancer [61]. Moreover, only one study revealed curcumin’s role in regulating the expression of miR-7-5p, and this study was conducted on patients with brain damage and cognitive dysfunction disease [62].

Furthermore, the significant association of CKS2, TOP2A, and PARP1 identified through PPI analysis highlights the potential importance of these genes in developing BC treatment strategies targeting the hsa_circ_0001946/miR-7-5p axis. Consistent with the findings in the literature, *CKS2* expression was found to be elevated in luminal BC patient tissue samples and cell lines. Additionally, Western blot analysis demonstrated a significant decrease in CKS2 protein levels in BC cell lines following curcumin treatment. CKS2 belongs to the mammalian cyclin kinase subunit (CKS) family, which has two members (CKS1 and CKS2). CKS2 is involved in cell proliferation and cell cycle regulation and plays an important role in somatic cell division. CKS2 is an oncogene that is aberrantly expressed in various malignant tumor tissues, including BC, and is closely associated with various biological processes such as tumor development, progression, and metastasis [36,63,64]. Increased expression has been associated with large tumor size, a lack of PR expression, and poor survival. Therefore, it is thought that the *CKS2* gene may serve as a good prognostic biomarker for BC patients [65]. Huang et al. showed that *CKS2* inhibition significantly reduced BC cell proliferation and invasiveness and led to a significant reduction in the mean weight and volume of tumors in nude mice [66]. Taken together, our results and data from other studies suggest that *CKS2* may serve as a potential oncogenic biomarker and therapeutic target in BC. Moreover, the association between miR-7-5p and the *CKS2* gene has been demonstrated only in a study conducted on papillary thyroid carcinoma tissues and cell lines. In that study, it was reported that the upregulation of miR-7-5p inhibited cell proliferation, invasion, and migration by targeting *CKS2*, suggesting that the miR-7-5p/*CKS2* axis could serve as a foundation for developing miRNA-targeted therapies in papillary thyroid carcinoma [36].

Considering the results altogether, curcumin shows promise in cancer therapy due to its anti-inflammatory, antioxidant, and anticancer effects. Research in this area is steadily increasing in order to further enhance the anticancer activity of curcumin [7]. Nevertheless, systematic validation with appropriate randomized clinical trials is required to enable translation from the bench to bedside. In addition, several curcumin-related challenges need to be overcome. Due to its rapid metabolism, poor absorption, low water solubility, and tendency to cause gastrointestinal discomfort in high oral doses, its effectiveness in clinical applications is limited. The encapsulation of curcumin in various nanostructures has been shown to improve its bioavailability and therapeutic effects. For example, lipid-based nanoparticles with a neutral surface charge provide good mucus permeability and prolonged lipolysis, thereby enhancing curcumin bioavailability. Moreover, the use of nanostructures with hydrophilic properties helps to overcome the solubility issue. It is also worth noting that modifying nanoparticle surfaces to specifically target tumor cells has been shown to enhance the therapeutic efficacy of curcumin [9,47,67]. The intravenous delivery of curcumin-encapsulated nanoparticles improves absorption and clinical outcomes compared to oral delivery. Although intravenous administration provides superior bioavailability and allows for the direct targeting of tumor cells, it is more invasive and costly than oral administration [48]. Despite its benefits, some side effects of curcumin, including headaches, diarrhea, and nausea, should also be taken into account in cancer treatment [67]. At present, curcumin is used as a dietary supplement but is not regarded as an approved drug. The Food and Drug Administration (FDA) has not approved curcumin for the treatment of cancer or any other medical condition [68]. Ongoing investigations into curcumin’s role in BC are vital for enhancing therapeutic efficacy and improving patients’ quality of life.

In this preliminary study, curcumin demonstrated significant anticancer effects in BC cell lines at 10 µM for 24 h. While these data provide mechanistic insight, translating in vitro concentrations into clinical applications remains difficult. Clinical research has reported encouraging findings with different curcumin formulations and doses. For instance, intravenous curcumin (300 mg weekly for 12 weeks) combined with paclitaxel improved safety and efficacy in patients with advanced BC [52]. In another randomized clinical trial, nanocurcumin capsules at 80 mg daily for six months reduced anthracycline-induced cardiotoxicity in BC patients [69]. A phase II clinical study conducted over five months in 52 BC patients reported that 2% curcumin gel effectively alleviated radiation dermatitis and improved patient quality of life during radiotherapy [70]. Moreover, oral curcumin at 500 mg daily for six months prevented tamoxifen-induced nonalcoholic fatty liver disease in ER+ patients without adverse effects [71]. Collectively, these findings highlight curcumin’s therapeutic promise, but further in vivo studies and clinical trials are required to validate these results.

In summary, this preliminary study provides novel insights into the molecular mechanisms by which curcumin may exert its anticancer effects in BC. By demonstrating that curcumin modulates the hsa_circ_0001946/miR-7-5p/*CKS2* axis, we uncover a previously uncharacterized circRNA-mediated regulatory pathway involved in BC progression. These findings not only support the therapeutic potential of curcumin but also emphasize the emerging role of circRNAs as key modulators and potential biomarkers in cancer biology. Nevertheless, further validation using in vivo models, additional BC subtypes, and larger clinical cohorts is necessary to confirm the translational relevance of our findings. Future in vitro research should include rescue experiments (e.g., overexpression of hsa_circ_0001946 or inhibition of miR-7-5p) and focus on the development of advanced delivery systems to enhance the bioavailability of curcumin, thereby optimizing its clinical efficacy. Moreover, the possibility of off-target effects from siRNA and miRNA mimic experiments cannot be completely excluded, as they may influence other genes and cellular pathways beyond the intended targets. Although appropriate controls were used, future studies will employ rescue experiments to confirm specificity. Although CKS2 was validated at the protein level, other differentially expressed genes could not be confirmed due to budgetary and resource constraints; subsequent studies will aim to expand protein-level validation to additional targets.

## 5. Conclusions

Our preliminary findings suggest that curcumin may suppress BC growth by regulating the hsa_circ_0001946/miR-7-5p/target gene axis. Specifically, curcumin downregulates hsa_circ_0001946, upregulates miR-7-5p, and reduces the expression of key oncogenic targets such as *CKS2*, thereby inhibiting cell proliferation and migration while promoting apoptosis in BC cells. These results uncover a novel molecular mechanism through which curcumin exerts its anticancer effects and highlight the hsa_circ_0001946/miR-7-5p/*CKS2* axis as a promising therapeutic target. Although restricted to in vitro and in silico findings, our study further suggests that this circRNA–miRNA–gene regulatory network may serve as a clinically relevant biomarker for early detection, molecular subtype classification, and the development of precision-targeted therapies in BC.

## Figures and Tables

**Table 1:** Forward and reverse primer sequences of the studied genes.

Gene	Primer Sequences	Reference
***CDR1as***	F 5′-ACGTCTCCAGTGTGCTGA-3′	[19]
	R 5′-CTTGACACAGGTGCCATC-3′	
***GAPDH***	F 5′-GCCATCAATGACCCCTTCAT-3′	[35]
	R 5′-TGACAAGCTTCCCGTTCTCA-3′	
***CKS2***	F 5′-TTCGACGAACACTACGAGTACC-3′	[36]
	R 5′-GGACACCAAGTCTCCTCCAC-3′	
***GINS1***	F 5′-AGGTCACTGGGAGGAGATGA-3′	[37]
	R 5′-TCGAGGTAAAAAGTGCTGGCT-3′	
***TOP2A***	F 5′-CATTGAAGACGCTTCGTTATGG-3′	[38]
	R 5′-CCAGTTGTGATGGATAAAATTAATCAG-3′	
***NREP***	F 5′-TTGAGCGAATGCTACCAGAG-3′	[39]
	R 5′-AGGCGAGGCTACGGAAAG-3′	
***SLC7A5***	F 5′-TAGGAGACAGAGCCAAGCAC-3′	[40]
	R 5′-CACGGGAACAACAGAAACAA-3′	
***PARP1***	F 5′-CCACACACAATGCGTATGACT-3′	[41]
	R 5′-CCACAGCAATCTTCGGTTATGA-3′	
***TMEM97***	F 5′-CTCCAAAGCCAGTGGTTTC-3′	[42]
	R 5′-GCCAGTGGTTGTTTCCTTC-3′	
***UHRF1***	F 5′-TGTCAAGGGTGGCAAGAAT-3′	[43]
	R 5′-GCCAGGCTCATCATCGTC-3′	
***CANT1***	F 5′-GCTTCTCGTCCTTCAAGTTCATC-3′	[44]
	R 5′-ACGCTTCCGATCTTGGTCTC-3′	
***MARCKSL1***	F 5′-CAGGCTACAGAGCCATCCACTC-3′	[45]
	R 5′-GCAGCTTAGAGATCACCCACCT-3′	

F: forward primer; R: reverse primer.

**Table 2:** Demographic characteristics of patients.

Characteristics	Number (%)
**All**	65 (100.0)
**Age, mean ± SD**	56.45 ± 11.89
**Age group**	
≤50	22 (33.8)
>50	43 (66.2)
**Menopausal status**	
Premenopausal	20 (30.8)
Postmenopausal	45 (69.2)
**Family history of BC**	
Yes	16 (24.6)
No	49 (75.4)
**Smoking**	
Yes	13 (20.0)
No	52 (80.0)
**Alcohol consumption**	
Yes	3 (4.6)
No	62 (95.4)

SD: Standard deviation.

**Table 3:** Clinicopathological characteristics of patients.

Characteristics	Category	Number (%)
**Molecular subtype**	Luminal A	30 (46.2)
	Luminal B	35 (53.8)
**Histological Type**	Invasive ductal carcinoma	47 (72.3)
	Invasive lobular carcinoma	9 (13.8)
	Mixed	5 (7.7)
	Other	4 (6.2)
**Pathological tumor stage**	I	17 (26.2)
	II	46 (70.8)
	III	2 (3.1)
**Pathological lymph node involvement**	Negative	38 (58.5)
	Positive	27 (41.5)
**Histological tumor grade**	I	4 (6.2)
	II	33 (50.8)
	III	28 (43.1)
**Tumor focal status**	Unifocal	52 (80.0)
	Multifocal	13 (20.0)
**Lympho-vascular invasion**	Yes	39 (60.0)
	No	26 (40.0)
**Estrogen receptor**	Negative	0 (0.0)
	Positive	65 (100.0)
**Progesterone receptor**	Negative	6 (9.2)
	Positive	59 (90.8)
**HER2**	Negative	59 (90.8)
	Positive	6 (9.2)
**Ki-67 expression**	<20%	27 (41.5)
	≥20%	38 (58.5)
**Surgical treatment-Breast**	Breast-conserving surgery	53 (81.5)
	Mastectomy	12 (18.5)
**Surgical treatment-Axilla**	Sentinel lymph node biopsy	57 (87.7)
	Axillary lymph node dissection	8 (12.3)

## Data Availability

The original contributions presented in this study are included in the article. Further inquiries can be directed to the corresponding author.

## Abbreviations

The following abbreviations are used in this manuscript:

BC	Breast Cancer
CircRNA	Circular RNA
MiRNA	MicroRNA
ncRNAs	Non-Coding RNAs
HG-DMEM	High-Glucose Dulbecco’s Modified Eagle Medium
DMSO	Dimethyl Sulfoxide
NT siRNA	Non-Target siRNA Negative Control
NT-miRNA	Non-Target miRNA Mimic Negative Control
PBS	Phosphate-Buffered Saline
qPCR	Quantitative Real-Time PCR
PPI	Protein–Protein Interaction
IC50	Half-Maximal Inhibitory Concentration
DNMT	DNA Methyltransferase
5-Aza-2′-Deoxycytidine	5-Azacytidine and Decitabine
CKS2	Cyclin Kinase Subunit 2
